# Genome-wide transcriptional analysis of 14-3-3 genes in *Paulownia fortunei* under abiotic and biotic stresses

**DOI:** 10.1186/s12870-026-09001-y

**Published:** 2026-06-12

**Authors:** Pingluo Xu, Haibo Yang, Mengmeng Ji, Xin Mao, Guoqiang Fan

**Affiliations:** Institute of Paulownia, Henan Agricultural University, Zhengzhou, 450002 Henan P.R. China

**Keywords:** *Paulownia fortunei*, 14-3-3 protein family, Abiotic stresses, Biotic stresses, Mitogen-Activated Protein Kinase cascade

## Abstract

**Background:**

The 14-3-3 proteins are evolutionarily conserved regulators in eukaryotes and play pivotal roles in plant stress responses and immunity. *Paulownia* is a fast‑growing tree valued for its high‑quality timber and diverse industrial applications, but it encounters multiple abiotic and biotic stresses, including drought, salinity, and phytoplasma‑induced witches’ broom disease. To better understand the molecular mechanisms underlying stress resilience in *Paulownia fortunei*, a comprehensive analysis of the 14-3-3 gene family was conducted.

**Results:**

Genome‑wide identification revealed 14 *Paulownia fortunei* (*P. fortunei*) *general regulatory factor* (*PfGF*) genes, which were phylogenetically classified into ε and non‑ε groups, each exhibiting distinct exon‑intron structures and stress‑responsive cis‑elements in their promoters. Transcriptome and quantitative real‑time PCR analyses demonstrated their differential expression under drought, salt, and phytoplasma infection. Notably, *PfGF10* was upregulated under drought, *PfGF2* showed salt‑specific induction, while *PfGF7* was downregulated in phytoplasma‑infected axillary buds, suggesting its involvement in witches’ broom disease progression. Molecular docking, yeast two‑hybrid, and bimolecular fluorescence complementation assays confirmed that PfGF7 interacts with *Paulownia fortunei* receptor‑like kinase a (RLKa), receptor‑like kinase b (RLKb), and mitogen‑activated protein kinase kinase kinase A (MAPKKKA); similarly, *PfGF11* interacts with the same three partners. Subcellular localization in *Nicotiana benthamiana* leaves revealed that PfGF7, PfGF11, PfRLKa, and PfRLKb localize to the cell membrane. Furthermore, co‑expression of PfGF7 or PfGF11 with PfRLKa, PfRLKb, or PfMAPKKKA in *N. benthamiana* leaves significantly induced cell death and upregulated the expression of defense genes including NbPR1a, NbLOX, and NbERF1.

**Conclusions:**

Our findings suggest that *Paulownia fortunei* PfGF proteins modulate the MAPK signaling pathway, potentially regulating immune responses through their interactions with receptor‑like kinases and MAPKKKA. This study provides crucial insights for cultivating stress‑resistant *Paulownia* varieties and advancing sustainable forestry practices.

**Supplementary Information:**

The online version contains supplementary material available at 10.1186/s12870-026-09001-y.

## Introduction

14-3-3 proteins are evolutionarily conserved eukaryotic regulators [1], also known as General Regulatory Factor (GFs) or G-box factor 14-3-3 homolog proteins (GF14). These plant proteins typically function as homo- or heterodimers within the G-box complex, functioning as dimeric phosphoserine-binding proteins. Each monomer within these dimers is capable of interacting with diverse proteins, conferring upon them unique functional attributes [2–5]. By recognizing phosphorylated sites on target proteins, 14-3-3 proteins bind to them, thereby modulating the stability, subcellular localization, or interactions with other proteins of these targets [2, 3, 6, 7]. Consequently, they play pivotal regulatory roles in ion channel function, hormonal signaling pathways, and responses to abiotic stress, as well as in growth and development processes [8–11].

During the growth and development of plants, 14-3-3 proteins are essential regulators of fundamental plant developmental processes including cell division, differentiation, elongation, and apoptosis, by interacting with various signal transduction proteins [12–14]. When plants encounter abiotic stresses such as water deficit, salinity, and high temperatures, they must activate a series of defense mechanisms to cope [10]. One way in which 14-3-3 proteins contribute to this response is by regulating hormonal signaling pathways. For instance, they bind to related proteins in the abscisic acid (ABA) signaling to regulate plant adaptation to these stresses [14]. For example, when the *Hv14-3–3 A* gene in barley was silenced, the stomatal density on the leaf surface increased, resulting in reduced water use efficiency and decreased drought tolerance [15]. Conversely, under drought stress, overexpression of the apple (*Malus domestica*) 14-3-3 gene *MdGF11* significantly alleviated the adverse effects of drought by activating stress-responsive gene expression [16]. This, in turn, reduced stomatal aperture and water loss rate, thereby enhancing drought tolerance [16]. Thus, by binding to various target proteins and forming stable complexes, 14-3-3 proteins regulate cellular physiology and metabolism, which is essential for plants to adapt to changing and often stressful environmental conditions.

14-3-3 proteins are key regulators of plant pathogen defense mechanisms [17]. In *Arabidopsis* (*Arabidopsis thaliana*), 14-3-3λ and 14-3-3κ can interact with multiple Receptor-like Cytoplasmic Kinases (RLCKs) and Mitogen-Activated Protein Kinase Kinase Kinase (MAPKKKs), facilitating the phosphorylation of MAPKKKs and the activation of the MAPK cascade, thereby enhancing plant disease resistance [18]. In tomato (*Solanum lycopersicum* L.), the expression of mRNAs for *PTI5*,* GRAS4*,* WRKY28* and *LRR22*, induced by *Xanthomonas campestris pv. vesicatoria (Xcv)*, requires the participation of *SlTFT1*. Upon recognition of effectors AvrTo and AvrToB, the NLR receptor complex Prf/Pto in tomato splits into dual ETI signaling pathways branches, with the SlTFT3-dependent branch fully activating the MAPK cascade, which is essential for subsequent inhibition of photosystem II and the expression of plant immune-related genes [17]. Similarly, multiple 14-3-3 proteins have been reported to participate in disease resistance in rice (*Oryza sativa* L.). For example, the rice bacterial blight effector XopN interacts with OsGF14d and OsGF14e to inhibit plant defense mechanisms [19, 20]; OsGF14c positively regulates *Rice* resistance to blast disease by modulating the stability of the OsSCL7 protein; and OsGF14f positively regulates rice resistance to bacterial blight and blast diseases through a salicylic acid-dependent pathway [21]. Therefore, 14-3-3 proteins serve as molecular scaffolds and play a crucial role in plant immune regulation.

Native to China, *Paulownia* is a fast-growing deciduous tree valued for its high-quality wood and aesthetic appeal, has a wide range of industrial applications. Maturing in just six years, it holds significant economic and ecological importance [22–24]. However, it is susceptible to various abiotic (e.g., low temperatures, drought, salinity, heavy metal ions) and biotic stresses (e.g., phytoplasma infection), which can severely impact yield and economic benefits [22, 25]. *Paulownia* witches’ broom (PaWB) disease, caused by phytoplasma, is characterized by symptoms including excessive shoot proliferation, dwarfing, yellowing, and phyllody. This disease severely reduces timber quality and yield, leading to substantial economic losses in *Paulownia* plantations [23]. Although the genome of *Paulownia* has been well characterized and the 14-3-3 gene family is known to play critical roles in stress responses across many species, the regulatory functions of 14-3-3 proteins in woody plant growth, development, and immunity remain poorly understood. To date, only limited reports have addressed this family in *Paulownia*. Therefore, this study conducted a genome-wide identification of the *14-3-3* gene family in *Paulownia*, identifying 14 genes. This study comprehensively analyzed their physicochemical properties, phylogeny, gene structures, synteny, promoter elements, expression patterns, and protein interactions. This study also validated the interaction between PfGFs and their target proteins to explore their mechanism of response to phytoplasma infection. This study provides new insights into improving the stress resistance of *Paulownia* and offers valuable references for developing more stress‑resistant forest germplasm resources and promoting sustainable forestry development.

## Materials and methods

### Identification and chromosomal localization of *PfGFs*

The *P. fortunei* genome sequences were retrieved NCBI (https://www.ncbi.nlm.nih.gov) and obtain the 14-3-3 protein domain HMM profile (PF00244) from the Sanger database, which is used to retrieve sequences from the *Paulownia* genome and to remove redundant sequences. To ensure completeness, a BLASTP search with *Arabidopsis* GRFs protein sequences (e-value ≤1e-3) was conducted. After removing redundancies, the protein domain integrity was verified using CDD Pfam and SMART. The high-confidence, non-redundant genes were named as *P. fortunei* 14-3-3 (PfGF), based on pseudomolecule positions. Prediction of physicochemical properties and chromosome distribution refer to Yang (2025) [26].

### Motif, conservative domain and gene structure analysis of *PfGFs*

The MEME suite (https://meme-suite.org/meme) was used to identify 10 conserved motifs in the PfGF protein sequences. The conservative motifs and protein structure prediction of PfGFs refer to Yang (2025) [26].

### Phylogenetic analysis of PfGFs

Protein sequences of 14-3-3s from *Arabidopsis thaliana*, *Malus domestica*, *Mangifera indica* and *P. fortunei* were aligned using MEGA12 (v 12.1) [27] software. Subsequently, the NJ method was used to build the phylogenetic tree with 1000 bootstrap replicates.

### Cis acting element analysis of *PfGFs* gene family

TBtools (v 2.453) [28] was used to extract the promoter region 2000 base pairs upstream of the transcription start site of the *PfGFs* gene. The PlantCARE database (http://bioinformatics.psb.ugent.be/webtools/plantcare/html/) was used to analyze promoter sequences and predict cis-acting elements.

### Expression pattern analysis of the *PfGFs* under biotic and abiotic stress conditions

*P. fortunei* (PF) plantlets were propagated from a single clone maintained in the Institute of *Paulownia* of Henan Agricultural University. For salt stress treatment (PF044), 30‑day‑old in vitro plantlets were cultured under controlled conditions (25 ± 2 °C, 130 µmol·m⁻²·s⁻¹ light intensity, 16 h light/8 h dark photoperiod). The plantlets were then transplanted to outdoor conditions for 30 days, after which uniformly growing individuals were selected and planted singly in nutrient pots containing equal amounts of ordinary garden soil. After 50 days of normal growth, salt stress was applied by irrigating the soil with 200 mmol·L⁻¹ NaCl solution. Each treatment group contained three biological replicates.

For drought stress treatment (PFH), uniformly growing PF plantlets were planted singly in nutrient pots with equal amounts of soil. After 10 days of normal growth, the plants in the drought group were subjected to water withdrawal, while the control group continued to receive normal watering. After 9 days of drought stress, the second pair of leaves from the shoot apex were collected from both control and drought‑treated plants, immediately frozen in liquid nitrogen, and stored at -80 °C for RNA extraction.

For pathogen stress, phytoplasma‑infected *P. fortunei* (PFI) plantlets showing typical witches’ broom symptoms were used. Infected plantlets were treated with 60 mmol·L⁻¹ methyl methanesulfonate (MMS) for 5, 10, and 20 days, designated as PFIM60‑5, PFIM60‑10, and PFIM60‑20, respectively. All samples were collected and stored as described above.

Using the coding sequences (CDS) of *PfGF* genes obtained from the *Paulownia* genome, we investigated their expression patterns under drought, salt, and phytoplasma infection. Transcriptomic data collected before and after each stress treatment were analyzed to identify differentially expressed *PfGF* genes. Based on the FPKM (Fragments Per Kilobase Million) values, we generated heatmaps using TBtools (v 2.453) to visualize the dynamic expression profiles of *PfGF* genes across different stress conditions.

### Real-time fluorescence quantification (qRT-PCR)

To validate transcriptome data, Total RNA was extracted using the Plant RNA Extraction Kit (TIANGEN BIOTECH (BEIJING) Co., LTD., Beijing, China). Extract total RNA from *paulownia* tissue-cultured seedlings and perform DNase I treatment to eliminate DNA contamination. cDNA synthesis was performed using the PrimeScript RT Reagent Kit (TAKARA, Japan). Quantitative real-time PCR (qRT-PCR) was conducted on an Applied Biosystems™ 7500 Fast Real-Time PCR System (Thermo Fisher Scientific, USA), with the Actin gene as an internal reference. All qRT-PCR data were analyzed using GraphPad Prism (v 8.0.2), with statistical significance determined by one-way ANOVA (*P* < 0.05 considered significant), and results are presented as mean ± SD from three independent biological replicates; the same software was used for figure generation. The primers related to qRT-PCR are provided in Supplementary Table S1.

### Molecular docking analysis

To elucidate the molecular basis of the interactions between PfGFs and their potential ligands, we performed molecular docking simulations. Initially, the molecules of interest were thoroughly prepared by dehydrating and hydrogenating them using AutoDock Tools (v 1.5.7) [29] software. These optimized molecules were then exported in separate files, designated as ligands and receptors, respectively. For the docking process, we utilized AutoDock Vina (v 1.2.5), a powerful tool that simulates the binding of ligands to receptors based on energy minimization principles. The binding interactions were quantitatively evaluated by assessing the binding affinity values, which provide a measure of the strength of the interaction. Furthermore, we conducted statistical analysis of the binding energies to ensure the robustness of our findings. Finally, the results of the molecular docking simulations were visually represented using PyMOL (v 3.0), allowing us to analyze the structural features and interaction patterns of the PfGFs and their ligands.

### Subcellular localization and cell death assay

Gene cloning of *PfGF7*,* PfGF11*,* PfRLKa*,* PfRLKb* and *PfMAPKKKA* was performed using KOD DNA polymerase, and the pRI101 expression vector containing a green fluorescent protein (GFP) tag was ligated through homologous recombination. The obtained recombinant plasmids 35 S: pRI101-*PfGF7*-eGFP, 35 S: pRI101-*PfGF11*-eGFP, 35 S: pRI101-*PfRLKa*-eGFP, 35 S: pRI101-*PfRLKb*-eGFP, and 35 S: pRI101-*PfMAPKKKA*-eGFP were transformed into GV3101-Psoup-P19 and transiently expressed in *N. benthamiana* leaves. After culturing transformed plants at 25℃ for 72 h, eGFP signals were visualized and captured using a laser confocal microscope (A1HD25, Nikon, Tokyo, Japan). For cell death assays, *N. benthamiana* leaves were infiltrated with Agrobacterium carrying the indicated constructs, with Agrobacterium carrying INF1 used as a positive control. Twenty‑four hours later, leaves were further infiltrated with Agrobacterium harboring *INF1*. Cell death in the agroinfiltrated areas was examined 4 d post-agroinfiltration and the dead cells were stained with trypan blue. Primers related to gene cloning are listed in Supplementary Table S1.

### Yeast two-hybrid assay

To identify proteins interacting with each of the two PfGF family members, we used the STRING database (v 11.5) [30] to predict potential interactions and construct a protein interaction network. To validate these predictions, we performed a yeast two‑hybrid assay by co‑transforming recombinant pGADT7 and pGBKT7 vectors into yeast AH109 cells. The negative control consisted of the empty pGADT7 vector co‑transformed with the recombinant pGBKT7 vector. Transformed yeast were plated on SD/-Trp/-Leu medium and incubated at 30 °C for 2–3 days. Colonies were further screened on SD/-Trp/-Leu/-Ade/-His medium with X‑α‑Gal to confirm protein interactions based on reporter gene expression [26]. Primers related to gene cloning are listed in Supplementary Table S1.

### Bimolecular fluorescence complementation (BiFC) assay

Agrobacterium GV3101-Psoup-P19 carrying the BiFC vectors was resuspended and adjusted to an OD600 of 0.3–0.5. Then introduce BiFC carrier, mix thoroughly and incubate for 2 h to ensure effective formation of the complex. Subsequently, the transformed *Agrobacterium* was infused into tobacco leaves using a needle-free syringe. After 48 h, fluorescence was observed using a laser confocal microscope (A1HD25, Nikon, Tokyo, Japan). All confocal images were acquired using a microscope with a 20× eyepiece. For fluorescence detection, the EGFP channel was excited at 488 nm and emission collected at 500–550 nm; the mCherry channel was excited at 561 nm and emission collected at 570–620 nm. The Gain value was set to 91, laser intensity to 3.7, and pinhole to 2.4 Airy units. These settings were kept constant across all samples to ensure comparability. Primers related to gene cloning are listed in Supplementary Table S1.

### Data availability

The datasets generated and/or analysed during the current study are available in the [NCBI] repository, [GenBank: GCA_019321725.1 (*P. fortunei* Genome) and PRJNA624264 (RNA-Seq data)]. The BioProject accession PRJNA221355 (BioSample: SAMN02363446) corresponds to the drought stress treatment, and PRJNA289582 (BioSample: SAMN03856042) corresponds to the salt stress treatment (200 mmol·L^− 1^ NaCl). The *P. fortunei* used in this study came from the Forest Biotechnology Laboratory of the Institute of Paulownia, Henan Agricultural University, Zhengzhou, China., and permission has been granted to collect *P. fortunei*.

## Results

### Genome-wide identification and characterization of 14-3-3 family genes in *Paulownia*

The *P. fortunei* genome contains 14 putative 14-3-3 genes, designated PfGF1 through PfGF14 according to their chromosomal positions (Table 1). All members of the PfGF family possess a conserved 14-3-3 domain characteristic of 14-3-3 proteins. Table 1 summarizes the physicochemical features of the PfGF protein family. The coding sequences of *PfGFs* range in length from 738 bp to 879 bp, encoding proteins varying from 245 to 292 amino acids. These proteins exhibit molecular weights (MWs) of 27.65–33.21 kDa and isoelectric point (pI) values between 4.70 and 5.05, with GRAVY indices ranging from − 0.682 to -0.224,indicating their hydrophilic nature. The aliphatic index values vary from 37.39 to 80.96, suggesting that some PfGFs are intrinsically unstable. Subcellular localization predictions reveal that the 14-3-3 proteins of *P. fortunei* are targeted to the nucleus, nucleocytoplasmic, chloroplast, Golgi apparatus, and peroxisome. Notably, 50% of PfGFs are predicted to locate at the nucleocytoplasmic membrane, while 21.4% are predicted to be at the plasma membrane, potentially playing roles in cell membrane recognition. Additionally, PfGF6 is localized to the peroxisome, hinting at its involvement in plant stress resistance.

**Table 1:** Physical and chemical properties of *Paulownia fortunei* GF family

Gene id	Gene name	Length(aa)	MW(KDa)	Pi	GRAVY	Aliphatic index	Subcelluar location	Formula
Pfo06g002470.1	*PfGF1*	255	28.64	4.75	-0.483	85.02	Nuclear/Plasma membrane	C_1253_H_1992_N_336_O_412_S_9_
Pfo06g002710.1	*PfGF2*	245	27.65	4.7	-0.224	37.39	Plasma membrane	C_1225_H_1939_N_317_O_387_S_11_
Pfo06g008390.1	*PfGF3*	261	29.69	4.78	-0.39	46.89	Nuclear/Plasma membrane	C_1315_H_208_7N_343_O_421_S_8_
Pfo07g003600.1	*PfGF4*	259	29.45	4.82	-0.682	48.69	Nuclear	C_1278_H_2023_N_347_O_426_S_12_
Pfo09g014000.1	*PfGF5*	292	33.21	4.82	-0.368	44.66	Golgi Apparatus	C_1473_H_2314_N_382_O_468_S_11_
Pfo11g008980.1	*PfGF6*	267	30.44	4.9	-0.493	49.32	peroxisome	C_1342_H_2126_N_360_O_428_S_9_
Pfo11g009090.1	*PfGF7*	261	29.4	4.74	-0.521	46.32	Chloroplast	C_1286_H_2041_N_349_O_420_S_9_
Pfo13g001630.1	*PfGF8*	255	28.67	4.74	-0.527	45.61	Nuclear/Plasma membrane	C_1251_H_1986_N_338_O_414_S_9_
Pfo13g001840.1	*PfGF9*	245	27.75	4.82	-0.242	41.73	Plasma membrane	C_1229_H_1952_N_322_O_385_S_11_
Pfo13g006090.1	*PfGF10*	282	32.23	5.05	-0.39	47	Nuclear/Plasma membrane	C_1425_H_2246_N_382_O_447_S_11_
Pfo15g009150.1	*PfGF11*	260	29.5	4.72	-0.54	43.63	Nuclear/Plasma membrane	C_1293_H_2052_N_348_O_421_S_9_
Pfo15g009330.1	*PfGF12*	255	29.2	4.92	-0.613	51.42	Plasma membrane	C_1282_H_2025_N_351_O_412_S_8_
Pfo16g003150.1	*PfGF13*	256	29.02	4.72	-0.573	50.66	Nuclear/Plasma membrane	C_1262_H_2001_N_341_O_422_S_10_
Pfo17g003170.1	*PfGF14*	260	29.64	4.91	-0.656	49.04	Nuclear/Plasma membrane	C_1300_H_2057_N_345_O_425_S_10_

### Multiple sequence alignment and phylogenetic analysis of *PfGFs*

**Fig. 1:**
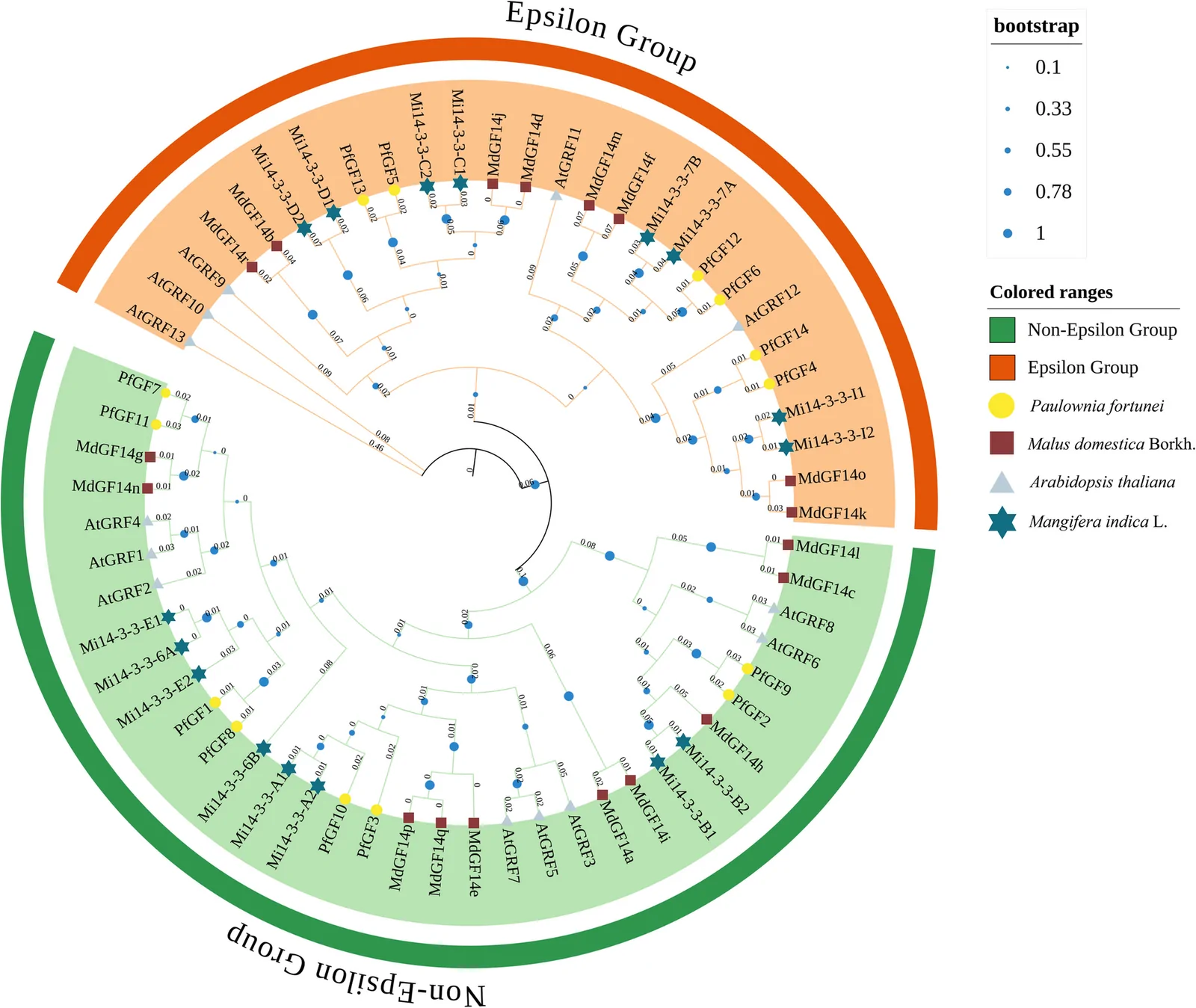
Phylogenetic analysis of *PfGF* genes in *P. fortunei*, *Arabidopsis thalian*,* Malus domestica* And *Mangifera indica*

Sequence alignment showed a high degree of similarity across all *P. fortunei PfGF* family members, with similarity percentages ranging from 59.27% to 99.62%, indicating relatively high sequence conservation. Despite the highly conserved structure of this family, considerable variations were observed in the N- and C-termini of the 14-3-3 proteins during multiple sequence alignment and conserved motif analysis. To investigate the evolutionary relationships within the *Paulownia* 14-3-3 family, a phylogenetic tree was constructed using the amino acid sequences of *Paulownia* PfGFs, *A. thaliana* AtGRFs, *Malus domestica* Borkh. MdGF14s and *Mangifera indica* L. Mi14-3–3s. The phylogenetic analysis showed that, similar to the subclassification of other 14-3-3 gene family subtypes, Paulownia PfGFs were classified into non-Epsilon (ε) and ε groups, with the non-ε group containing 8 PfGFs, 8 AtGRFs, 10 MdGF14s, and 8 Mi14-3–3s members, and the ε group comprising 6 PfGFs, 5 AtGRFs, 8 MdGF14s, and 8 Mi14-3–3s members (Fig. 1). Analysis of sequence similarity and divergence among *Paulownia* 14-3-3 protein family members, as well as the construction of the phylogenetic tree with AtGRF, MdGF14 and Mi14-3-3 proteins, suggests that these proteins have evolved conservatively across different plant species.

### Gene structure and conserved motif analysis of *PfGFs*

**Fig. 2:**
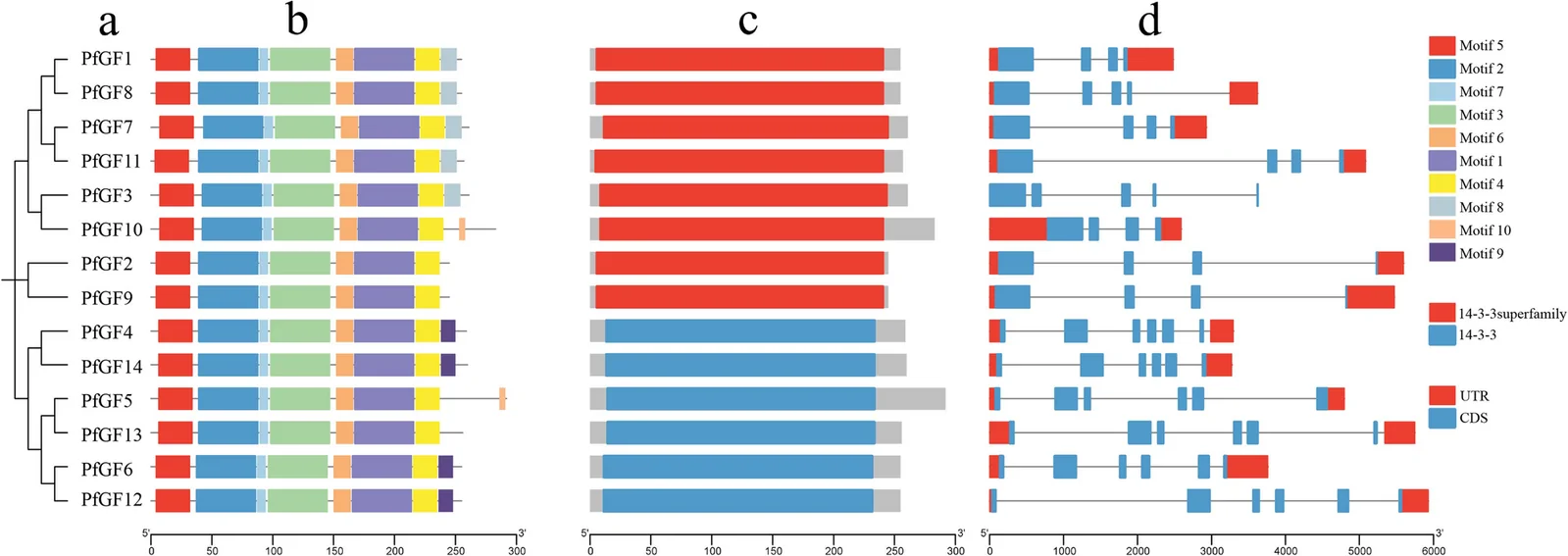
Evolutionary relationships (**a**), motifs (**b**), conserved domain (**c**) and gene structures (**d**) between the PfGFs members in *P. fortunei*

Based on the phylogenetic tree of PfGF proteins, eight PfGFs (PfGF1/2/3/7/8/9/10/11) belong to the non-ε group, while six PfGFs (PfGF4/5/6/12/13/14) belong to the ε group, and both groups have their own unique 14-3-3 conserved domains (Fig. 2a). MEME analysis revealed 10 conserved motifs in PfGF proteins, with motifs 1–7 present in all ε and non-ε members. Notably, motif 9 was uniquely found in PfGF5 and PfGF10, while C-terminal regions exhibited subfamily-specific variations influencing target protein specificity.Additionally, motif 8 is predominantly specific to non-ε PfGF proteins (Fig. 2b). By comparing genomic and coding sequences, the exon-intron structures of *PfGF* genes were obtained. All *PfGF* genes in the ε group have six introns, while *PfGF* genes in the non-ε group contain 0–3 introns (Fig. 2c). This motif-based classification aligns with phylogenetic grouping. (Fig. 2d).

### Cis-element analysis of *PfGFs* gene promoters

**Fig. 3:**
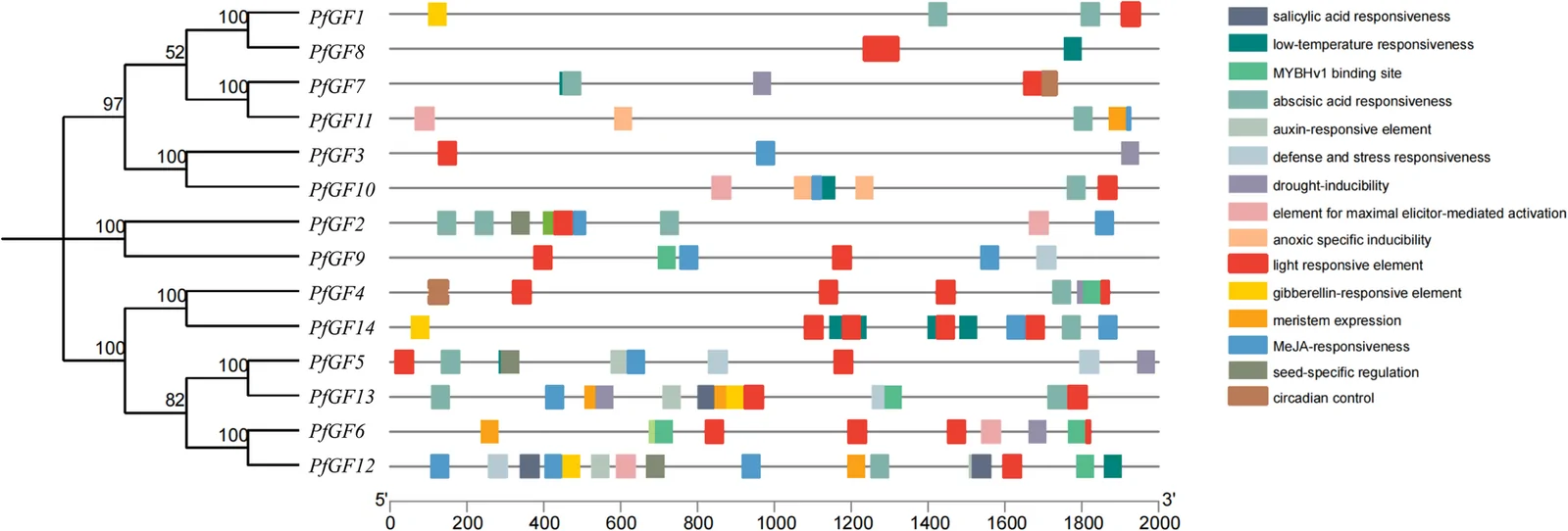
Analysis of cis-acting elements in the promoter region of the *PfGFs* in *P. fortunei*

This study examined their promoter regions to investigate expression patterns and regulatory characteristics of *Paulownia 14-3-3* genes. (Fig. 3). Our findings revealed that *PfGF* gene promoters abound with cis-acting elements linked to hormones and stress responses. Specifically, this study identified 17 hormone-related cis-elements, including ABREs for abscisic acid, AuxRR-core and TGA-element for auxin, CGTCA-motif for MeJA, P-box and TATC-box for gibberellin, ERE for ethylene, and TCA-element for SA. Gibberellin-responsive elements are mainly in *PfGF12*, *PfGF13*,* PfGF14* (ε group) and *PfGF1* (non-ε group). Auxin-responsive elements appear in *PfGF4*,* PfGF5*,* PfGF12*,* PfGF13* (ε group) and *PfGF7* (non-ε group). Salicylic acid-responsive elements are exclusive to *PfGF12* and *PfGF13*. Most *PfGF* genes, including *PfGF1*,* PfGF2*,* PfGF4*,* PfGF5*,* PfGF7*,* PfGF10*,* PfGF11*,* PfGF12*,* PfGF13*, and *PfGF14*, contain ABREs. All *PfGF* genes except *PfGF1*,* PfGF4*,* PfGF7*, and *PfGF8* possess MeJA-responsive elements. These findings hint at *PfGF* genes’ potential roles in hormone responses. Additionally, we identified five types of stress-related cis-elements (ARE, LTR, MBS, TC-rich repeats, and W-box). Except for *PfGF1*,* PfGF2*, and *PfGF4*, all *PfGF* genes have at least one to two stress-related cis-elements, suggesting their responsiveness to various environmental stresses.

### *PfGF* family evolution: chromosomal localization and cross-species homology

**Fig. 4:**
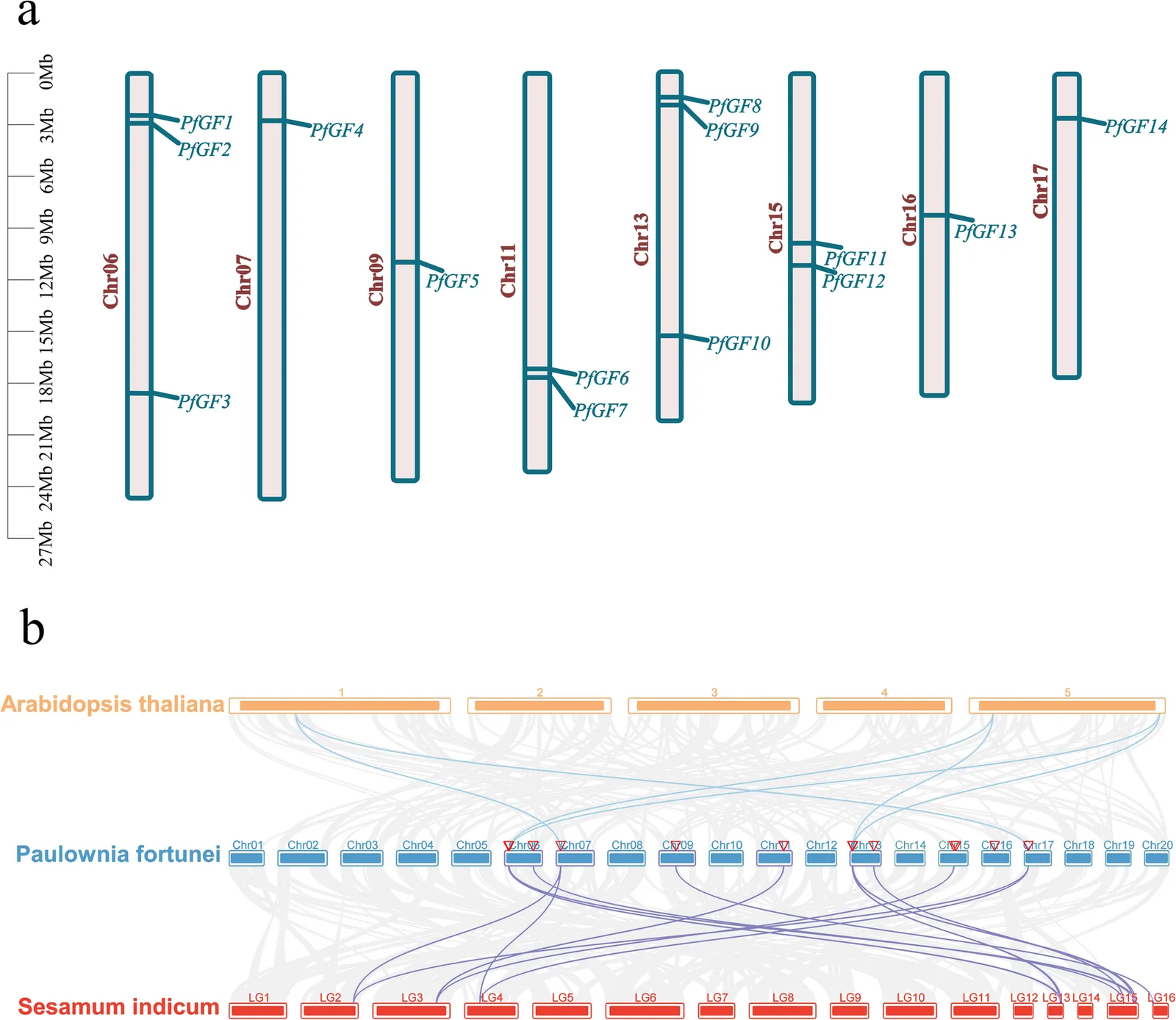
Chromosome distribution and collinearity analysis of the *PfGFs* genes in *P. fortunei*. **a** Distribution of *PfGF* genes in chromosomes, **b** Collinearity analysis of *PfGF* genes between *P. fortunei* and *A. thaliana*, *P. fortunei* and *S. indicum*

The chromosomal localization analysis (Fig. 4a) indicates that *PfGF* family members are unevenly distributed across 17 *Paulownia* chromosomes. Notably, chromosomes 6 and 13 harbor the highest density of these genes (three per chromosome), contrasting with the remaining six chromosomes carrying one to two *PfGF* members each, which collectively reflects a balanced genomic arrangement. Comparative synteny studies further highlight distinct evolutionary patterns: while six homologous gene pairs are conserved between *Paulownia* and *Arabidopsis* (Fig. 4b), alignment with *Sesame* (*Sesamum indicum* L.) reveals a substantial expansion to 14 syntenic pairs (Fig. 4b). This enhanced homology suggests closer phylogenetic proximity between *Paulownia* and *Sesame* within the *PfGF* family, potentially attributable to ancestral lineage conservation or recent interspecific genetic exchange events.

### Expression patterns of *PfGFs* gene under abiotic stress conditions

**Fig. 5:**
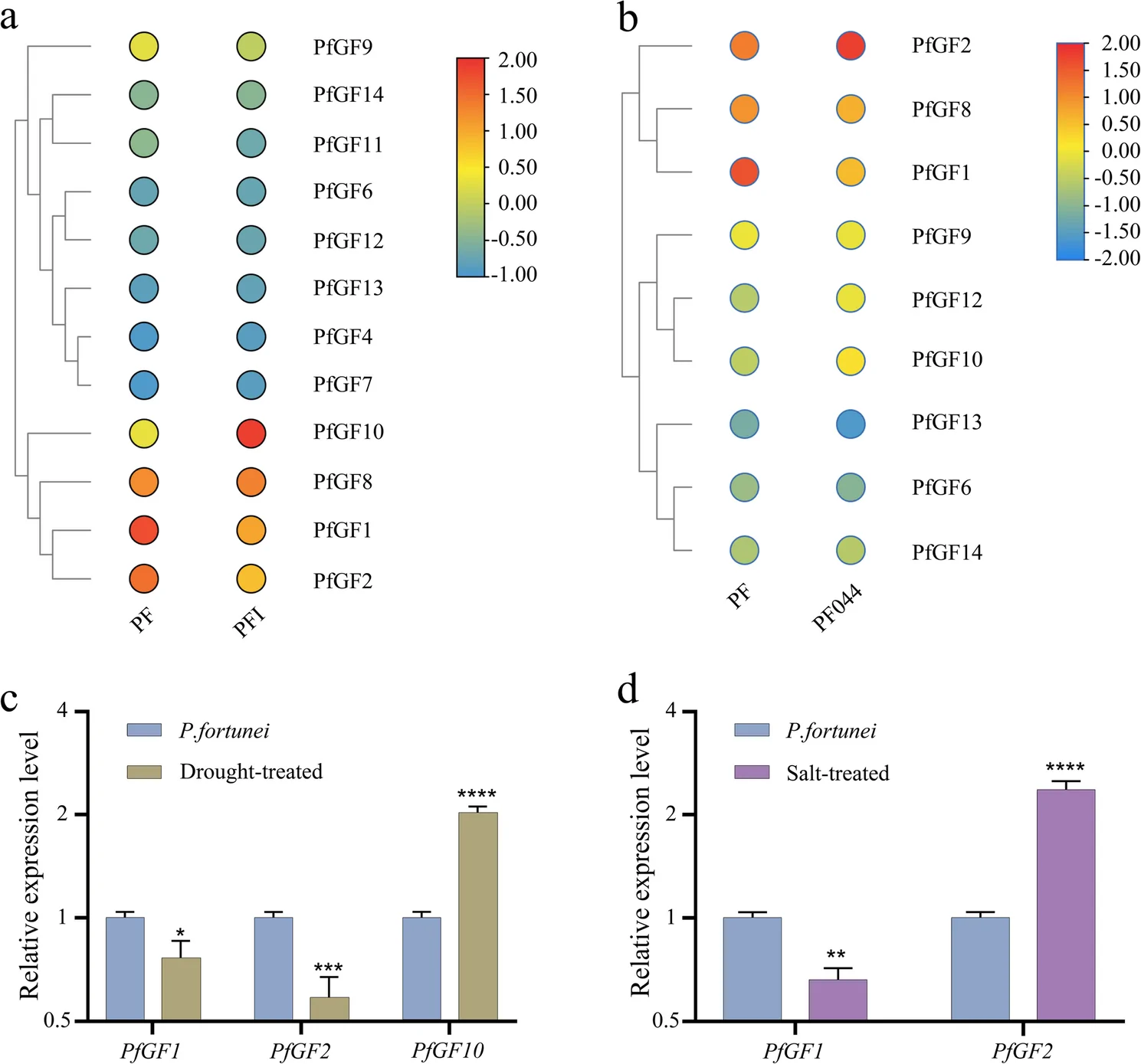
Expression profiles of *PfGF* genes in *Paulownia fortunei* under drought (**a**) and salt (**b**) stress; qRT-PCR detection of expression of *PfGF* genes under pathogen drought (**c**) and salt (**d**) stress. PF: *Paulownia fortunei* (control); PFH: *P. fortunei* under drought stress; PF044: *P. fortunei* under salt stress

An extensive analysis of the transcriptional responses of 14 *PfGF* genes was conducted in *Paulownia* under drought (PFH) and 200 mmol·L⁻¹ NaCl salt stress conditions (PF044) (Fig. 5). Under drought stress, a clear trend emerged: the transcription levels of *PfGF1* and *PfGF2* were significantly downregulated. Conversely, *PfGF10* showed a marked upregulation, suggesting a potential adaptive role under drought stress (Fig. 5a). In contrast, under salt stress, the transcription of *PfGF2* was significantly enhanced, indicating its potential role in regulating salt stress response in *Paulownia*. However, the expression level of *PfGF1* decreased significantly, pointing to downregulation in response to salt stress (Fig. 5b). To validate the RNA‑seq results, the expression patterns of selected *PfGF* genes under drought and salt stress were examined by qRT‑PCR using three independent biological replicates. As shown in Fig. 4, the qRT‑PCR results were highly consistent with the transcriptomic data. Under drought stress, the transcript levels of *PfGF1* and *PfGF2* were significantly downregulated, whereas *PfGF10* was significantly upregulated compared with control conditions (Fig. 5c). Under salt stress (200 mmol·L⁻¹ NaCl), *PfGF1* showed significant downregulation, while *PfGF2* exhibited significant upregulation (Fig. 5d). These results confirm the reliability of the RNA‑seq expression profiles and support the conclusions drawn from the transcriptomic analysis. Together, these findings highlight the complex and specific regulatory roles played by individual *PfGFs* in the stress response mechanisms of *Paulownia*.

### Biotic stress-induced differential expression of PfGF genes

**Fig. 6:**
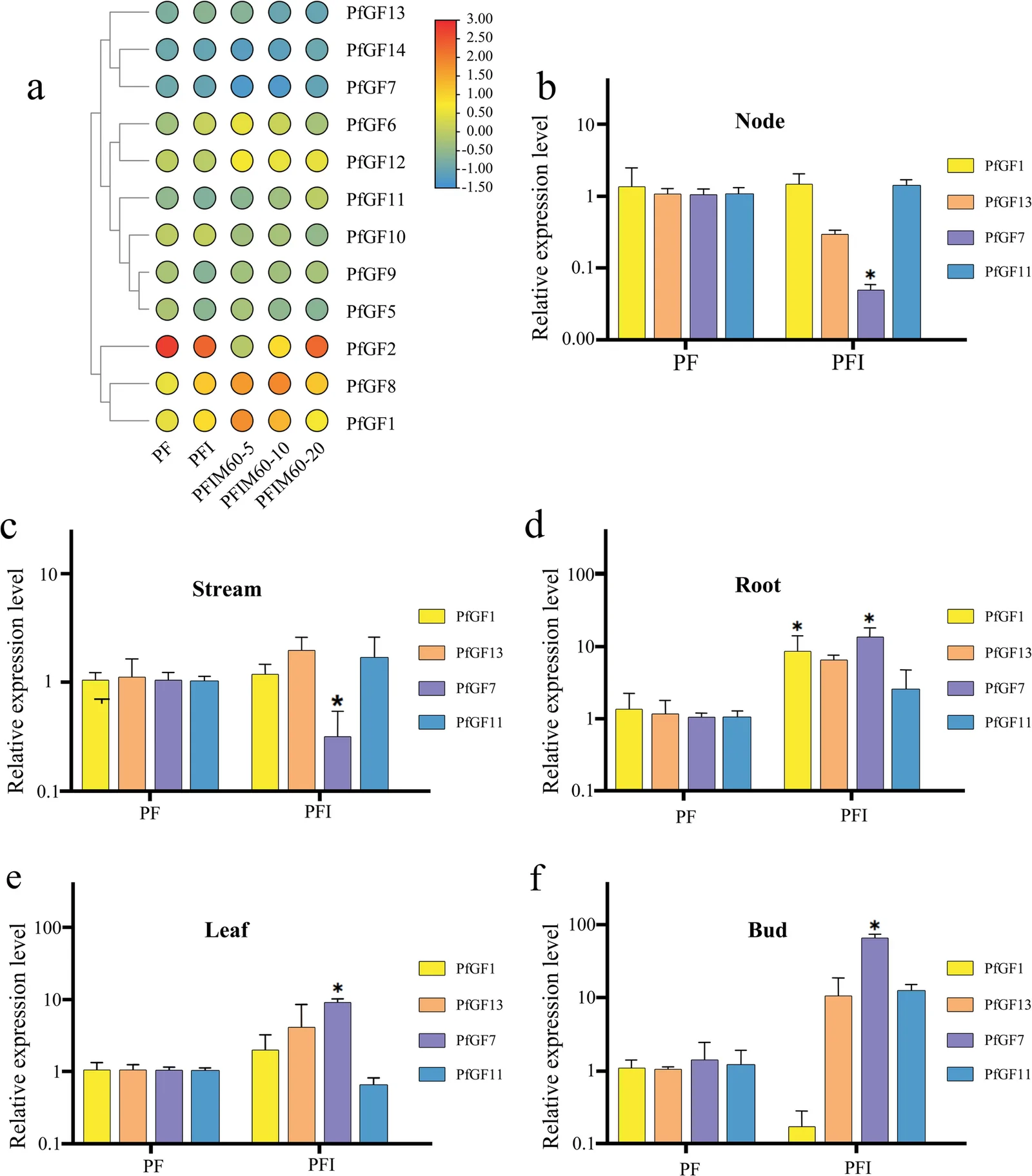
Expression profiles of *PfGF* genes in *Paulownia fortunei* under phytoplasma stress. **a** Expression of *PfGF* genes in the occurrence of *Paulownia* witches’ broom disease; **b**-**f** qRT-PCR detection of expression of *PfGF* genes in different tissues under pathogen stress. PF: *Paulownia fortunei* (control); PFI: *Paulownia* witches’ broom phytoplasma-infected *P. fortunei*; PFIM60-5/10/20: infected plants treated with MMS for 5, 10, and 20 days, respectively

To explore the transcriptional responses of *Paulownia PfGF* genes to phytoplasma infection, this study performed a transcriptome analysis. This revealed distinct gene expression patterns post-infection (Fig. 6a). Specifically, *PfGF8*,* PfGF1*,* PfGF6*,* PfGF14*,* PfGF13*,* PfGF7*,* and PfGF10* were upregulated, suggesting roles in plant defense. In contrast, *PfGF9* and *PfGF5* were downregulated, indicating suppression under stress. Treating infected seedlings with 60 mmol/L methyl methanesulfonate (MMS) for 5–10 days reduced the phytoplasma load, leading to complete recovery after 20 days. Transcriptome data showed initial downregulation followed by upregulation of *PfGF1*,* PfGF7*,* PfGF11*, and *PfGF13* with MMS treatment. qRT-PCR validated these findings (Fig. 6b-f, Supplementary Table S1), showing upregulation of four selected *PfGF* genes in most tissues of infected seedlings, except for *PfGF7*, which was downregulated in axillary buds—the disease sites (Fig. 6b).

### Tissue-specific expression dynamics of *PfGF* genes in *Paulownia*

Expression analysis of four *PfGF* genes in *Paulownia* tissues showed significant variation. Across leaves, stems, buds, nodes, and roots, distinct patterns emerged (Fig. 6b-f). *PfGF11* was stably expressed in all tissues before and after infection. *PfGF7* was highly expressed, especially in nodes, and downregulated in nodes post-infection, suggesting a role in axillary bud growth. *PfGF1* and *PfGF13* had low expression in healthy tissues but were upregulated in roots and leaves post-infection, respectively, hinting at roles in root biology and leaf development/stress responses. These findings highlight the diverse tissue-specific roles of *PfGFs* in *Paulownia* growth and development.

### Subcellular localization of PfGF7 and PfGF11

**Fig. 7:**
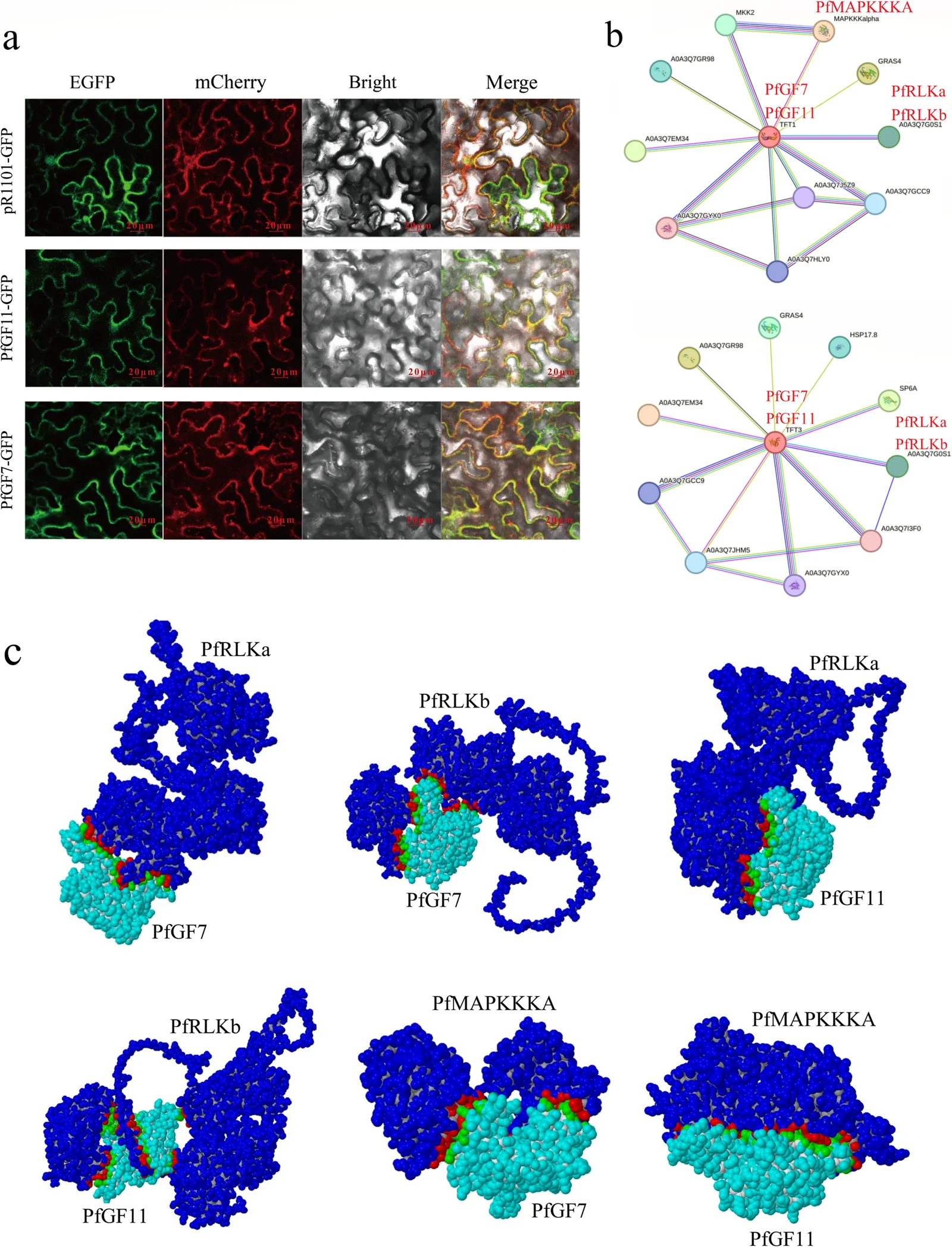
The interaction between PfGFs and candidate interacting proteins. a: Subcellular localization of PfGF7-GFP and PfGF11-GFP in plant cells. GFP, green fluorescent protein. mCherry, plasma membrane marker (pSuper1300-mCherry-OsMCA1), bar: 20 μm; b: Prediction of protein interactions in PfGFs through string database; c: Predicting the interaction between PfGFs and interacting proteins through molecular docking

Given the unique expression pattern of PfGF7 in *Paulownia* infected with phytoplasma, further analysis of both PfGF7 and PfGF11 was conducted. To verify their subcellular localization, transient expression vectors for PfGF7 and PfGF11 were constructed and expressed in *Nicotiana benthamiana* leaves. Results indicated co-localization of PfGF7-GFP and PfGF11-GFP fluorescent signals with a plasma membrane marker (pSuper1300-mCherry-OsMCA1) (Fig. 7a). Therefore, we conclude that PfGF7 and PfGF11 play regulatory roles at the cell membrane in response to phytoplasma infection.

**Table 2:** Hydrogen bond interaction, interface area and binding activation energies between PfGFs and PfRLKs or PfMAPKKKA

Structure	interface area, Å2	△G, kcal/mol
PfGF7 + PfRLKa	2546.1	-26.5
PfGF7 + PfRLKb	1379.5	-11.8
PfGF11 + PfRLKa	2059.9	-22.2
PfGF11 + PfRLKb	1389.1	-16
PfGF7 + PfMAPKKKa	2089.1	-18.1
PfGF11 + PfMAPKKKa	2278.8	-12.2

### Interaction Analysis of PfGF7 and PfGF11

**Fig. 8:**
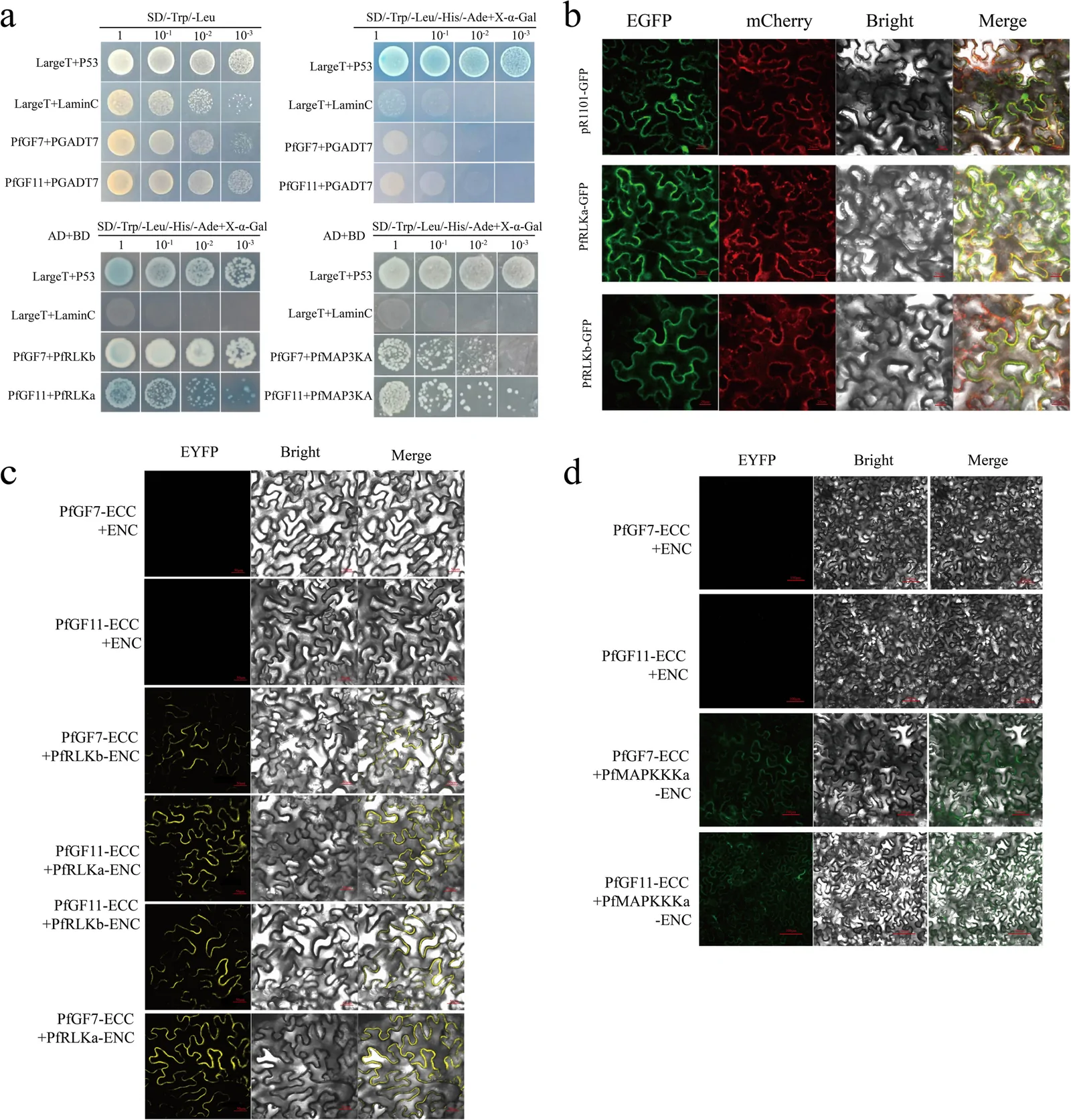
Analysis and verification of interacting proteins in PfGFs. **a** Verification of the interaction between PfGFs and interacting proteins using Y2H; **b** Subcellular localization of PfRLKa-GFP and PfRLKb-GFP in plant cells. mCherry, plasma membrane marker (pSuper1300-mCherry-OsMCA1), bar: 20 μm; **c**, **d** Verification of the interaction between PfGFs and interacting proteins using BiFC, Channel: EYFP, bar: c, 50 μm, d, 100 μm

To further investigate the functions of *Paulownia* PfGF7 and PfGF11, we first predicted their protein interaction networks using the STRING database, revealing interactions with protein kinases and MAPKKK from the MAPK cascade (Fig. 7b). We then screened for and identified homologs in *Paulownia*, naming them PfRLKa, PfRLKb, and PfMAPKKKA, respectively. To validate these interactions, yeast two-hybrid experiments were conducted. As demonstrated in Fig. 7a, yeast cells co‑transformed with the empty prey vector (pGADT7) and the bait vectors (pGBKT7-PfGF7 or pGBKT7-PfGF11) showed no growth on selective media, confirming the absence of autoactivation. In contrast, co-transformed yeast colonies exhibited robust growth on SD/-Trp/-Leu/-Ade/-His medium with X-α-Gal-induced blue coloration, validating specific interactions between PfGF7-PfRLKb, PfGF11-PfRLKa, PfGF7-PfMAPKKKA, and PfGF11-PfMAPKKKA (Fig. 8a). The subcellular localization results in the leaves of *Nicotiana benthamiana* indicated that PfRLKa and PfRLKb were also localized on the cell membrane (Fig. 8b). To further verify the interactions between PfGFs and PfRLKs, as well as PfMAPKKKA, molecular docking analysis and bimolecular fluorescence complementation (BiFC) experiments were conducted. The molecular docking results revealed that the six complexes, each consisting of PfGF7/PfGF11 and PfRLKa/PfRLKb/PfMAPKKKA respectively, exhibited robust hydrogen bonding, which serves as the primary force driving their interactions (Fig. 7c; Table 2). BiFC yielded similar results, with PfGF7/11 in combination with PfRLKa/b respectively, producing strong YFP fluorescent signals at the cell membrane (Fig. 8c). Similarly, co-expression of PfMAPKKKA with PfGF7 and PfGF11 also resulted in YFP fluorescent signals at the cell membrane, while no such signals were observed in the control group (Fig. 8d).

### PfGF7 and PfGF11 induce cell death and the expression of defense genes in *N. benthamiana*

**Fig. 9:**
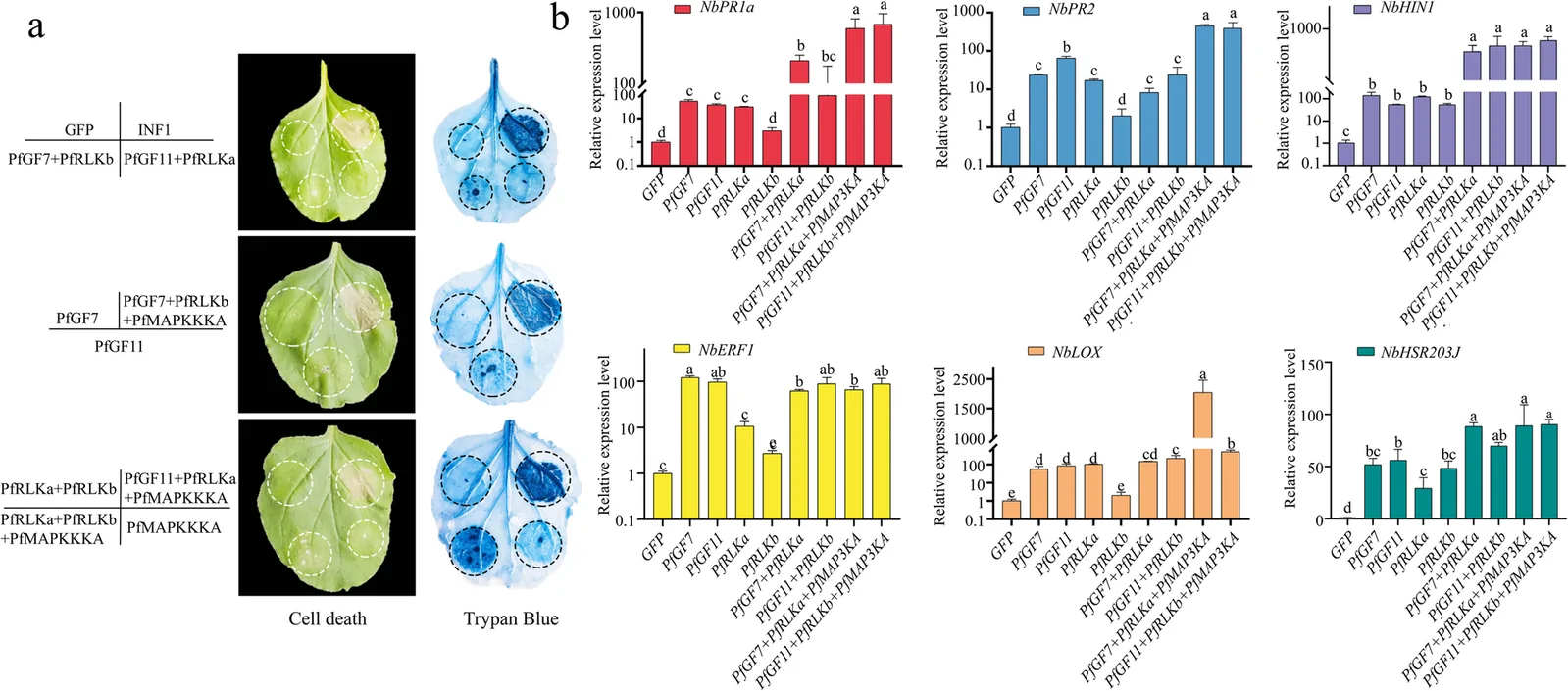
a: Cell death and Trypan blue-stained dead cells in *N. benthamiana* leaves transiently expressing or co-expressing *PfGFs*, *PfRLKs* and *PfMAPKKKA*,* INF1* as a positive control for cell death. The experiments in (**a**) were independently performed three times with similar results; **b** Expression changes of defense genes in *N. benthamiana* leaves transiently alone or in combination *PfGF7*, *PfGF11*, *PfRLKa*, *PfRLKb*, and *PfMAPKKKA* 48 h post-agroinfiltration. pRI101-GFP was used as a negative control. *NbGAPDH* was used as an internal reference. Different letters indicate significant differences (*p* < 0.05, one-way ANOVA)

The roles of PfGF7 and PfGF11 in plant immunity were investigated by analyzing cell death and defense gene expression in *N. benthamiana*. We transiently expressed each gene alone, as well as in various combinations with *PfRLKa*/b, and *PfMAPKKKA*, and *INF1* was transiently expressed as a positive control for cell death. It was found that transient expression of *PfGFs*, *PfRLKs*, or *PfMAPKKKA* alone induced only mild cell death. However, co-expression of all three genes triggered strong cell death comparable to that induced by *INF1* (Fig. 9a). Subsequently, qRT-PCR analysis was performed on the aforementioned transiently expressed samples. Compared to leaves expressing the empty vector (GFP) control, leaves individually expressing *PfGF7* or *PfGF11* exhibited significant upregulation in transcript levels of SA signaling-responsive genes *NbPR1a* and *NbPR2*, JA signaling gene *NbLOX*, ethylene signaling gene *NbERF1*, and hypersensitive response (HR) marker genes *NbHIN1* and *NbHSR203J* (Fig. 9b, Supplementary Table S1). However, co-expression of *PfGFs*, *PfRLKs*, and *PfMAPKKKA* induced substantially stronger expression of these defense genes in leaves (Fig. 9b). Moreover, leaves individually expressing *PfRLKa* or *PfRLKb* showed comparatively lower induction of defense gene expression (Fig. 9b). Therefore, *PfGF7* and *PfGF11* enhance immune responses in *N. benthamiana* leaves, while the co-presence of *PfRLKa/b* and *PfMAPKKKA* triggers a more potent immune activation.

## Discussion

The 14-3-3 proteins, initially discovered by Moore and Perez [31] in neural proteins extracted from bovine brain tissue, are present in various organelles in plants, including the nucleus, thylakoid membrane, mitochondria, and chloroplast stroma [32]. These proteins can form homodimeric or heterodimeric complexes, bringing different proteins into a single assembly [4, 5]. Consequently, they play pivotal roles in regulating multiple plant hormone signal transduction and metabolic processes, influencing plant growth, development, and stress responses [9, 10, 14]. In woody plants, the molecular mechanisms by which 14-3-3 proteins orchestrate the crosstalk between secondary growth and pathogen-induced immune responses are largely unexplored, Thus, for the first time, the identification of the 14-3-3 protein family and the validation of interacting proteins in *Paulownia* provide a new frontier for forest biotechnology. In this study, genomic characterization of *Paulownia* identified 14 *PfGF* genes, a number comparable to that of 14-3-3 gene family members in species such as *Arabidopsis* (15) [2, 33, 34], tomato (13) [35], grape (11) [36], rice (17) [37], mango (16) [38] and apple (18) [39], suggesting conservation of this gene family during plant evolution. Based on phylogenetic analysis, the *PfGF* gene family aligns with the classification of *Arabidopsis*, *Tomato*, and *Rice* species, divisible into ε group and non-ε group categories [33, 34]. Comparative gene structure analysis demonstrates that ε group *PfGF* genes in *Paulownia* exhibit higher exon-intron complexity compared to non-ε counterparts, a conserved feature shared by *Arabidopsis thaliana*, *Solanum lycopersicum*, and *Oryza sativa*, indicating potential evolutionary convergence in GF gene family organization. Conserved motif analysis showed that all identified *PfGF* genes possess seven conserved motifs, with variations in the C-terminal motifs influencing interactions between PfGF proteins and other proteins, leading to functional differentiation among family members. Additionally, the ε group and non-ε group *PfGF* genes in *Paulownia* share conserved domains with other species, further supporting the classification of 14-3-3 proteins. Collectively, the systematic delineation of the *PfGF* gene family is comprehensively validated through integrated phylogenetic reconstruction, exon-intron organization profiling, motif conservation screening, and structural domain alignment.

The crucial regulatory role of the 14-3-3 gene family in under different stress responses prompted us to analyze the transcriptional expression of *PfGFs* under drought and salt stress [10]. In *Rice*, overexpression of *OsGF14c* enhances drought tolerance in seedlings, whereas the mutant *osgf14b* exhibits greater drought tolerance compared to the wild type, suggesting a contrasting role for *OsGF14b* [40, 41]. Under drought stress, the *PcGF10* and *PcGF11* genes from the *Populus cathayana* 14-3-3 family are significantly induced [42]. In rice under salt stress, the interaction between OsGF14b and OsPLC1 (phospholipase C1) is promoted, and OsGF14b enhances the stability of OsPLC1 by inhibiting its ubiquitination, resulting in significantly increased salt tolerance in *OsGF14b*-overexpressing plants [43]. In this study, the non-ε group genes *PfGF2* and *PfGF10* are homologs of *OsGF14b* and *OsGF14c*, respectively. Under drought stress, the expression of *PfGF1* and *PfGF2* is downregulated, while *PfGF10* is significantly upregulated. In contrast, under salt stress, *PfGF2* expression is significantly upregulated, and *PfGF1* expression is significantly downregulated. Considering the homology between *PfGF2*, *PfGF10*, and *OsGF14b*, *OsGF14c*, combined with transcriptome analysis results, these genes may play different regulatory roles in different stress environments, making them promising target genes for stress-resistant breeding in *Paulownia* and even forestry.

Recent advances have elucidated the core regulatory mechanisms through which 14-3-3 proteins orchestrate the RLCK-MAPK signaling hub in plant immunity [44]. For instance, *Arabidopsis* 14-3-3 isoforms AtGRF6 and AtGRF8 engage in direct interactions with MAPKKK5, MEKK1, and RLCK VII subfamily members. These interactions disrupt the autoinhibitory effect of MAPKKK5’s N-terminal domain on its C-terminal region, enabling RLCK-mediated phosphorylation of the C-terminus. This phosphorylation cascade subsequently activates MAPK signaling downstream of PRRs, ultimately enhancing *Arabidopsis* resistance against bacterial and fungal pathogens [18]. In *Tomato*, the NLR receptor complex Prf/Pto dissociates into two ETI signaling branches upon recognition of pathogen effectors, where the phosphorylation of MAPKKKAα by Pto necessitates the direct mediation of *Tomato* 14-3-3 proteins TFT3 and TFT1, which is essential for fully activating the downstream MAPK cascade. In this study, PfGF7 and PfGF11 are homologs of TFT3 [17]. Our transcriptome data indicate that the expression of *PfGF1*,* PfGF7*,* PfGF11*, and *PfGF13* regularly increases and then decreases as the phytoplasma titer gradually decreases. Interestingly, qRT-PCR analysis of different tissue parts of *Paulownia* samples infected (PFI) and non-infected (PF) with phytoplasma revealed that only *PfGF7* was significantly downregulated in axillary buds, the disease site of PFI samples, suggesting that phytoplasma infection may suppress plant immunity by inhibiting the expression of *PfGF7*. In *Tomato*, TFT3 and TFT1 exhibit functional redundancy, and PfGF7 and PfGF11 share high homology. Therefore, this study speculates that PfGF7 and PfGF11 have similar functions to TFT3 and TFT1, and validated their interacting proteins. Our interaction results show that both PfGF7 and PfGF11 can interact with the *Paulownia* receptor-like kinase protein PfRLKa and PfRLKb, and PfGF7 also interacts with PfMAPKKKa, indicating a conserved regulatory mechanism. PfGF7 and PfGF11 may functionally redundantly mediate the phosphorylation of PfMAPKKKa by PfRLKs, activate the downstream MAPK cascade, and thus positively regulate the immune response in *Paulownia*. Therefore, our study provides a molecular basis for identifying disease-resistant proteins in *Paulownia* and lays the foundation for breeding new disease-resistant varieties of *Paulownia*.

## Conclusions

This study systematically identified 14 *PfGFs* genes in *Paulownia* and characterized their evolutionary relationships, structural features, and expression dynamics under drought, salinity, and phytoplasma infection. The *PfGFs* exhibited distinct stress-responsive expression profiles, with *PfGF2*, *PfGF7*, *PfGF10*, and *PfGF11* emerging as key candidates in abiotic and biotic stress tolerance. Notably, PfGF7 and PfGF11-homologs of tomato TFT3/TFT1- interacted with receptor-like kinases (PfRLKa/b) and PfMAPKKKA, suggesting their involvement in modulating the MAPK cascade for immune responses. The downregulation of *PfGF7* in phytoplasma-infected axillary buds further supports its role in pathogen response. These findings provide a molecular foundation for improving *Paulownia’*s stress resilience and offer potential genetic targets for breeding strategies.

## Supplementary Information

Supplementary Material 1: Primer sequences.

## Data Availability

The datasets generated and/or analysed during the current study are available in the [NCBI] repository, [GenBank: GCA_019321725.1 (*P. fortunei* Genome) and PRJNA624264 (RNA-Seq data)]. The BioProject accession PRJNA221355 (BioSample: SAMN02363446) corresponds to the drought stress treatment, and PRJNA289582 (BioSample: SAMN03856042) corresponds to the salt stress treatment (200 mmol·L-1 NaCl).
