# Supplementary material for: Genome-wide transcriptional analysis of 14-3-3 genes in Paulownia fortunei under abiotic and biotic stresses
Source: BMC Plant Biol. 2026 Jun 12;26:1416. doi: 10.1186/s12870-026-09001-y (PMC13487923; doi:10.1186/s12870-026-09001-y)
Supplement: Supplementary file 1 [file 12870_2026_9001_MOESM1_ESM.docx]

| **Supplementary Table S1** Primer sequences | | |
| --- | --- | --- |
| Gene name | Forward primer (5' to 3') | Reverse primer (5' to 3') |
| *PfGF1* | ATCATCGCTACTTGGCGGAG | GTTGAGTGCTAGTCCCAGCC |
| *PfGF2* | CAGATCTAAGGTCGAGGCCG | TCCCAACCTTAAACTCCGCC |
| *PfGF7* | AATCTGCTCTCGGTTGCCTA | CGTCGCAGATGCTAGAGAGC |
| *PfGF10* | ACTCTCCAGATCGTGCTTGC | AGGGTGGGAATAGGACTTCAGA |
| *PfGF11* | CGTCACCACCATCAGGGAAT | GAAGCAGAGGGAATGAGCCG |
| *PfGF13* | CGATTGGGTTTGGCCTTGAAT | ATCAGAGGTCCATAGCGTGA |
| *PfActin* | AATGGAATCTGCTGGAAT | ACTGAGGACAATGTTACC |
| *NbPR1a* | CCGCCTTCCCTCAACTCAAC | GCACAACCAAGACGTACTGAG |
| *NbPR2* | AGGTGTTTGCTATGGAATGC | TCTGTACCCACCATCTTGC |
| *NbLOX* | AAAACCTATGCCTCAAGAAC | ACTGCTGCATAGGCTTTGG |
| *NbERF1* | GCTCTTAACGTCGGATGGTC | AGCCAAACCCTAGCTCCATT |
| *NbHIN1* | CCAACTTGAACGGAGCCTATTA | AGGCATCCAAAGAGACAACTAC |
| *NbHSR203J* | ACGCAGATTTCAACCGAGTAT | GCCAGTCGCATTGGAGATAA |
| *NbGAPDH* | AGCTCAAGGGAATTCTCGATG | AACCTTAACCATGTCATCTCCC |
| *pGBKT7-*  *PfGF7* | TCAGAGGAGGACCTGCATATGATGGCGCCGCGTGAGGAG | TCGACGGATCCCCGGGAATTCCTGTTGTCCATCTTCAGGTTTGG |
| *pGBKT7-*  *PfGF11* | TCAGAGGAGGACCTGCATATGATGGCGGAGGCGGCAGCG | TCGACGGATCCCCGGGAATTCGTGTTGTCCTTCTTCAGGTTTGG |
| *pGADT7-*  *PfRLKa* | GCCATGGAGGCCAGTGAATTCATGGATGCTAAATTTCTTCACTTTCTG | CAGCTCGAGCTCGATGGATCCACTGGTCGATATGCATGAGTTACC |
| *pGADT7-*  *PfRLKb* | GCCATGGAGGCCAGTGAATTCATGATGGGCTGTGGAGTTTTGA | CAGCTCGAGCTCGATGGATCCTCTACCCTCAGATTTTACCAGTTGTG |
| *pGADT7-*  *PfMAP3KA* | GCCATGGAGGCCAGTGAATTCATGCCAGCTTGGTGGGATAA | CAGCTCGAGCTCGATGGATCCCTATGGCATGGTTCTTGTTGGTG |
| *pRI101-*  *PfGF7* | TCTTCACTGTTGATACATATGATGGCGCCGCGTGAGGAG | GCCCTTGCTCACCATGGATCCCTGTTGTCCATCTTCAGGTTTGG |
| *pRI101-*  *PfGF11* | TCTTCACTGTTGATACATATGATGGCGGAGGCGGCAGCG | GCCCTTGCTCACCATGGATCCGTGTTGTCCTTCTTCAGGTTTGG |
| *pRI101-*  *PfRLKa* | TCTTCACTGTTGATACATATGATGGATGCTAAATTTCTTCACTTTCTG | GCCCTTGCTCACCATGGATCCACTGGTCGATATGCATGAGTTACC |
| *pRI101-*  *PfRLKb* | TCTTCACTGTTGATACATATGATGATGGGCTGTGGAGTTTTGA | GCCCTTGCTCACCATGGATCCTCTACCCTCAGATTTTACCAGTTGTG |
| *pNC-BiFC-*  *PfGF7-Ecc* | AGTGGTCTCTGTCCAGTCCTATGGCGCCGCGTGAGGAG | GGTCTCAGCAGACCACAAGTCTGTTGTCCATCTTCAGGTTTGG |
| *pNC-BiFC-*  *PfGF11-Ecc* | AGTGGTCTCTGTCCAGTCCTATGGCGGAGGCGGCAGCG | GGTCTCAGCAGACCACAAGTGTGTTGTCCTTCTTCAGGTTTGG |
| *pNC-BiFC-*  *PfRLKa-Enc* | AGTGGTCTCTGTCCAGTCCTATGGATGCTAAATTTCTTCACTTTCTG | GGTCTCAGCAGACCACAAGTACTGGTCGATATGCATGAGTTACC |
| *pNC-BiFC-*  *PfRLKb-Enc* | AGTGGTCTCTGTCCAGTCCtATGATGGGCTGTGGAGTTTTGA | GGTCTCAGCAGACCACAAGTTCTACCCTCAGATTTTACCAGTTGTG |
| *pNC-BiFC-*  *PfMAP3KA-Enc* | AGTGGTCTCTGTCCAGTCCTATGCCAGCTTGGTGGGATAA | GGTCTCAGCAGACCACAAGTCTATGGCATGGTTCTTGTTGGTG |
